# Knockdown of Gasdermin D protects hippocampal neurons by regulating both pyroptosis and ferroptosis in a rat model of status epilepticus

**DOI:** 10.1186/s42494-026-00255-5

**Published:** 2026-05-04

**Authors:** Fan Feng, Haijiao Wang, Yanmei Tian, Rong Luo, Qianyun Cai

**Affiliations:** 1 Department of Pediatrics, West China Second University Hospital, Sichuan University, No. 20, Section 3, Renmin South Road, Chengdu, 610041 China; 2 Department of Pediatrics, Key Laboratory of Obstetric & Gynecologic and Pediatric Diseases and Birth Defects of the Ministry of Education, Sichuan University, Chengdu, 610041 China; 3 Department of Pediatrics, Key Laboratory of Development and Maternal and Child Diseases of Sichuan Province, Sichuan University, Chengdu, 610041 China

**Keywords:** Status epilepticus, GSDMD, Pyroptosis, Ferroptosis, Crosstalk

## Abstract

**Background:**

Pyroptosis and ferroptosis may be implicated in status epilepticus (SE)-induced neuronal damage. This study aimed to elucidate the effects of Gasdermin D (GSDMD) on pyroptosis and ferroptosis after SE, and to determine whether GSDMD inhibition ameliorates SE-induced neuronal damage and cognitive dysfunction.

**Methods:**

In the lithium-pilocarpine–induced SE rat model, we measured GSDMD and glutathione peroxidase 4 (GPX4) expression in the hippocampus via western blotting to evaluate the involvement of pyroptosis and ferroptosis after SE. After GSDMD knockdown by RNA interference, we performed Nissl staining to detect neuronal loss; quantitative reverse transcription polymerase chain reaction (qRT-PCR) to detect the hippocampal expressions of interleukin (IL)-1β, IL-6, and tumor necrosis factor-α (TNF-α); Prussian blue staining to detect iron deposition; and western blotting and enzyme-linked immunosorbent assay to detect the expressions of the ferroptosis-related molecules GPX4, malonaldehyde (MDA), and glutathione (GSH). Finally, cognitive and behavioral functions were evaluated using the spontaneous-activity test and novel object-recognition test.

**Results:**

Western blotting showed a marked increase in GSDMD expression (*P* < 0.050) and a notable decrease in GPX4 levels in (*P* < 0.050) the hippocampus of rats, providing evidence that both pyroptosis and ferroptosis are activated following SE. In addition, GSDMD knockdown significantly improved hippocampal neuronal survival and improved cognitive and behavioral functions of SE rats. Meanwhile, knockdown of GSDMD led to a significant reduction in the mRNA expression of IL-1β, IL-6, and TNF-α (pyroptosis products) (*P* = 0.021, 0.010, and 0.015, respectively), a decrease in iron deposition (*P* = 0.000) and MDA levels (ferroptosis product) (*P* = 0.000), and increased GSH and GPX4 expression (negative ferroptosis regulators) (*P* = 0.000 and 0.004, respectively).

**Conclusions:**

Pyroptosis and ferroptosis may jointly contribute to SE-induced neuronal damage. GSDMD may regulate both pyroptosis and ferroptosis, and inhibiting GSDMD could improve cognitive and behavioral function in SE rats.

**Supplementary Information:**

The online version contains supplementary material available at 10.1186/s42494-026-00255-5.

## Background

Epilepsy is a prevalent yet severe neurological condition, and status epilepticus (SE) refers to a single epileptic seizure of more than 30 min duration or a series of epileptic seizures during which function is not regained between seizures in a 30-min period [1]. SE is a neurological emergency. If it is not properly controlled, SE has the potential to cause permanent brain damage and even be life-threatening. Pathologically, it can result in severe neuronal damage, blood–brain barrier disruption, and hippocampal inflammation [2], all of which significantly increase disability and mortality rates among epilepsy patients. Even with active rescue, SE can progress to a chronic epileptic state, with 30–40% of patients experiencing concurrent cognitive impairment [3]. The precise mechanism of neuronal damage after SE remains incompletely understood, but recent research has demonstrated that regulated cell death (RCD) is a crucial factor in this process.

RCD is now defined as a collection of distinct cell death pathways triggered by the activation of various signal transduction mechanisms, which can be influenced by pharmacological or genetic interventions. Various RCDs have recently been confirmed to be associated with post-epileptic neuronal damage and seizure genesis [4, 5]. Therefore, the regulation of RCD has the potential to ameliorate neuronal death associated with SE and mitigate subsequent chronic seizures. Pyroptosis is a typical form of inflammatory RCD. Its molecular mechanism involves the activation of inflammasomes and the plasma membrane perforation mediated by Gasdermin family proteins. In a mouse model of epilepsy induced by pentylenetetrazole, up-regulation of Gasdermin D (GSDMD) -N protein and mRNA levels were observed after epilepsy. However, the administration of agmatine, an anti-inflammatory agent, effectively attenuated the expression of GSDMD-N, NLR family pyrin domain containing 3 (NLRP3) and interleukin (IL)−1β, blocked hippocampal neuronal damage and decreased seizure scores. The findings indicate that agmatine influences epilepsy development and provides neuroprotection by reducing neuroinflammation and inhibiting pyroptosis [6]. In addition, NLR family pyrin domain containing 1 (NLRP1) and NLRP3 inflammasome-mediated pyroptosis was also observed in the sclerotic hippocampal tissue of patients with temporal lobe epilepsy (TLE) [7]. Moreover, recent studies indicate that targeting pyroptosis signaling pathways, such as NLRP3 inflammasome and caspase-1, exhibit a certain efficacy in ameliorating epileptic seizures and cognitive impairment [8, 9].

The main characteristic of ferroptosis is the accumulation of iron-dependent lipid peroxides in cells, ultimately leading to oxidative stress-induced cell damage. It involves the following pathways: iron metabolism pathway, lipid metabolism pathway, glutathione (GSH)-dependent pathway, and mevalonate-dependent pathway. Ferroptosis has been implicated in both the onset and progression of epilepsy. Glutamic acid may serve as a predisposing factor for ferroptosis in epilepsy. During epileptic seizures, excess amounts of glutamic acid, an excitatory neurotransmitter, accumulate in the synaptic cleft. Elevated concentrations of extracellular glutamate can impede the activation of the cystine/glutamate reverse transporter (System Xc-), which is crucial for the GSH-dependent ferroptosis pathway, thereby indirectly leading to the reduced activity of glutathione peroxidase 4 (GPX4) and the increased buildup of lipid reactive oxygen species (ROS), ultimately causing ferroptosis [10]. In pentylenetetrazol-kindled and pilocarpine-induced epilepsy models, ferroptosis was proved to be involved in neuronal loss and seizure genesis. An increase in Ptgs2 mRNA, a marker of ferroptosis, was observed, along with a decrease in GSH levels and GPX4 protein expression, and a rise in malonaldehyde (MDA) and 4-hydroxy-2-nonenal (4-HNE) levels. Whereas, ferrostatin-1 can effectively relieve seizures by inhibiting ferroptosis [11]. Another study treated kainic acid (KA)-induced TLE rats with ferrostatin-1 and found that hippocampal synaptic plasticity was improved, thereby alleviating cognitive impairment [12].

Our previous experiments have also found a significant increase in key markers of pyroptosis and ferroptosis after SE. However, it has not yet been clearly determined whether pyroptosis and ferroptosis are individually or jointly involved in neuronal loss after SE, nor is the degree and importance of their involvement. Recent research has revealed that pyroptosis and ferroptosis share overlapping mechanisms and exhibit molecular crosstalk, although the specific mechanism remains unclear [13, 14]. Furthermore, the evidence linking pyroptosis and ferroptosis in epilepsy and SE is insufficient. Clarifying the underlying mechanism will contribute to mitigating neuronal damage, reducing hippocampal inflammation, and diminishing the risk of epileptogenesis, thereby presenting a new avenue for the treatment of SE.

Therefore, we employ the lithium-pilocarpine (Li-Pilo)-induced rat model to investigate the collaborative involvement of pyroptosis and ferroptosis in SE. Firstly, it is confirmed that both of pyroptosis and ferroptosis are activated in SE. Then, by knocking down GSDMD, we investigate the impact on pyroptosis as well as the neuroprotective effect. Thirdly, we study the influence of GSDMD knockdown on ferroptosis and explore the correlation between the two pathways. Finally, it will be determined whether knockdown of GSDMD plays a role in protecting neurons and improving cognitive function by regulation of both pathways of pyroptosis and ferroptosis.

## Methods

### Animals

In this study, specific pathogen-free Sprague–Dawley rats (age, 6–8 weeks; weight, 180–220 g; Sichuan Dashuo Animal Science and Technology Co. Ltd., Chengdu, China) were utilized. These rats were housed in a controlled environment with a 12-h light–dark cycle and were provided with free access to food and water. The room temperature was maintained at 22 °C ± 2 °C, and the humidity was kept at 55–58%. All experimental procedures were carried out in accordance with the National Institutes of Health's Guide for the Care and Use of Laboratory Animals [15]. In the spontaneous-activity tests and novel object-recognition tests, each group consisted of 4 rats; in other experiments, there were 3 rats per group. The study process was repeated three times for each sample in Western blotting and quantitative reverse transcription polymerase chain reaction (qRT-PCR); in other experiments, each sample underwent one experimental observation.

### Li-Pilo-induced SE model

To investigate the activation of pyroptosis and ferroptosis after SE, we randomly assigned the rats to the control (normal saline group, *n* = 6) and SE groups (*n* = 25). The rats in both groups were adaptively fed for 1 week. After this period, the rats in the SE group were intraperitoneally injected with 127 mg/kg lithium chloride (Sigma, St. Louis, MO, USA) followed by 45 mg/kg pilocarpine (Sigma, St. Louis, MO, USA) 18 h later to induce the SE model. Half an hour before the intraperitoneal injection of pilocarpine, the rats were given a 1-mg/kg intraperitoneal injection of atropine sulfate (Shanghai Maclin Biochemical Technology Co. Ltd., Shanghai, China) to alleviate peripheral cholinergic symptoms that might be caused by pilocarpine. Following the initial dose of pilocarpine, the rats were repeatedly administered pilocarpine intraperitoneally at a dosage of 10 mg/kg, at 30-min intervals. These injections were continued until the rats experienced SE that could be categorized as stage 4 or 5 on the Racine scale [16]. At 60 min after SE, 10 mg/kg diazepam (Shanghai Xudong Haipu Pharmaceutical Co. Ltd., Shanghai, China) was administered via intraperitoneal injection to terminate the attack. At all of the above time points, an equal volume of normal saline was administered to the rats in the control group.

### GSDMD knockdown SE rat model

The rats were mounted on a stereotaxic device and given an intraperitoneal injection of sodium pentobarbital at a dose of 50 mg/kg to induce anesthesia. After disinfection, the anterior fontanelle of the rats was exposed, and the hippocampus was located based on the coordinates of the brain atlas (anteroposterior: −5.3 mm; mediolateral: 4.0 mm; dorsoventral: −6.0 mm) [17]. A 0.5-μL injection of adeno-associated virus (AAV)-GSDMD-RNAi was gradually administered over 10 min at a rate of 0.05 μL/min into each hippocampal region. To reduce backflow along the injection route, the needle was left in place for 10 min after the injection was finished. The scalp was then sutured close using a surgical knot. The control rats were injected with AAV-Control-RNAi in the same way. At 1 week after the injections, the RNAi-treated rats were used to establish the Li-Pilo-induced SE model.

In order to examine the impact of GSDMD knockdown on pyroptosis and ferroptosis, we randomly divided the rats into the following 4 groups: (1) sham group (*n* = 30): stereotaxic injection of normal saline into the hippocampus, followed by intraperitoneal injection of normal saline after 1 week; and (2) SE group (*n* = 30), SE + AAV-Control-RNAi group (*n* = 30), and SE + AAV-GSDMD-RNAi group (*n* = 30): stereotaxic injection of normal saline, AAV-Control-RNAi, and AAV-GSDMD-RNAi into the hippocampus, respectively, followed by establishment of the SE model after 1 week. Behavioral tests were conducted on the rats on the 8th day of modeling, while other experiments were carried out on the 7th day.

### Western blotting

Protein levels were assessed using western blotting. All rats were administered a 1% solution of pentobarbital sodium and subsequently decapitated; both hippocampi of the rats were harvested immediately after death. The hippocampal samples were disrupted in radioimmunoprecipitation assay lysis buffer (Servicebio, Wuhan, China) and centrifuged at 12,000 × g for 30 min at 4 °C. Protein concentrations were determined using a bicinchoninic acid protein concentration assay kit (Beyotime, Shanghai, China). The proteins, with 30 μg per lane, were separated by 8% sodium dodecyl sulfate polyacrylamide gel electrophoresis and then transferred onto polyvinylidene fluoride (PVDF) membranes (Sigma, St. Louis, MO, USA) for further analysis. The membranes were incubated for 2 h with 5% skim milk diluted in Tris-buffered saline with Tween buffer on a shaker. After blocking, the PVDF membranes were incubated overnight at 4 °C with the appropriate primary antibodies: GSDMD (39754S, CST, Boston, MA, USA) and GPX4 (ab252833, Abcam, Cambridge, UK). β-Actin (AC026, Abclonal, Boston, MA, USA) was used as an up-sample control. After 3 washes with Tris-buffered saline containing Tween20, the PVDF membranes were incubated with the secondary antibody, horseradish peroxidase-labelled goat anti-rabbit antibody (s0001, Affinity, Jiangsu, China), for 2–3 h. Bands were visualized using enhanced chemiluminescence (zen-bio, Chengdu, China) and a chemiluminescence gel imager (Shanghai Tianneng Technology Co. Ltd., Shanghai, China). Strip analysis was performed using Tianneng GIS chassis control software (V2.0, Shanghai, China) to determine relative protein levels.

### Nissl staining

Hippocampal samples were first fixed in paraffin and then sectioned into 10-μm thick slices. The sections were deparaffinized and rehydrated through a gradient ethanol series, followed by incubation in a 1% toluidine blue solution (Servicebio, Wuhan, China) at 56 °C for 20 min. After staining, the slices were dehydrated in 95% ethanol, cleaned, and finally mounted with neutral balsam. The stained sections were examined using a digital trinocular camera microscope (BA210Digital, Xiamen, China). In each sample, 3 high-power fields at 400 × magnification were randomly selected. The Nissl-positive cells, which were recognized by significant levels of Nissl body, abundant cytoplasm, and a large cell body, were counted using the ImageJ software.

### Quantitative reverse transcription polymerase chain reaction

Hippocampal mRNA expression was determined using qRT-PCR. TRIzol reagent (Hefei Bomei Biotechnology Co. Ltd., Hefei, China) was used to extract the total RNA, and cDNA was generated using reverse transcription. The mRNA expression levels of IL-1β, IL-6, and TNF-α in the hippocampal neurons after SE were analyzed using their corresponding primers (β-actin: forward primer, 5′-GGGAAATCGTGCGTGACATT-3′ and reverse primer, 5′-GCGGCAGTGGCCATCTC-3′; IL-1β: forward primer, 5′-AATCTCACAGCAGCATCTCGACAAG-3′ and reverse primer, 5′-TCCACGGGCAAGACATAGGTAGC-3′; IL-6: forward primer, 5′-ACTTCCAGCCAGTTGCCTTCTTG-3′ and reverse primer, 5′-TGGTCTGTTGTGGGTGGTATCCTC-3′; TNF-α: forward primer, 5′-ATGGGCTCCCTCTCATCAGTTCC-3′ and reverse primer, 5′-CCTCCGCTTGGTGGTTTGCTAC-3′). The relative mRNA expression levels of IL-1β, IL-6, and TNF-α were calculated using the 2-△△CT method, with β-Actin as the reference.

### Prussian blue staining

Paraffin-embedded, 10-μm-thin hippocampal tissue sections were routinely dewaxed, rehydrated, stained with freshly prepared Prussian blue working solution at room temperature for 1 h, and then rinsed 3 times with distilled water. The slices were stained with nuclear solid red staining solution, soaked in distilled water for 10 min, dehydrated, sealed with neutral glue, and finally observed under a microscope. For each section, the entirety of the tissue sample was first observed at low magnification; then, 3 areas were randomly selected and photographed under high-power magnification (400 ×). Color threshold quantification was performed by ImageJ software. The average percentage of the positively stained area was calculated to quantify differences among different groups: percentage of positive expression area = positive expression area/visual field area (pixel area).

### Enzyme-linked immunosorbent assay

The GSH and MDA levels in the hippocampus were quantified using a Ray Biotech kit (Shanghai, China). Each well of a 48-well plate was supplemented with 50 μL of standard sample followed by 100 μL of horseradish peroxidase, and then incubated at 37 °C for 40 min. Next, each well was filled with washing solution, and washed 5 times. Subsequently, 50 μL of substrate solution A was added, followed by 50 μL of substrate solution B, and the samples were placed in an incubator in the dark at room temperature for approximately 20 min. Finally, 50 μL of termination solution was added to each well; the absorbance (optical density [OD] value) was measured at a wavelength of 450 nm by means of an enzymoleter within 15 min, and the concentrations of GSH and MDA in the sample were calculated.

### Spontaneous-activity test

The experimental device consisted of 2 parts: the spontaneous activity-detection box and the video-acquisition system. During the test, the experimental rats entered the spontaneous activity-detection box, and the number of grids crossed by the rats within 5 min was recorded. Before each rat was tested, the test chamber was wiped with 75% alcohol to prevent any odor left by the previous rat from interfering with the experimental results.

### Novel object-recognition test

The novel object-recognition test was conducted after the spontaneous-activity test. The experimental device used consisted of 2 identical black opaque plastic boxes and a video-capture system. The experimental process was divided into 3 stages: habituation, training, and detection. The novel object-recognition test lasted for 4 days. During the initial 2 days, i.e., the habituation stage, the rats were introduced to the test box in order to become accustomed to the environment in the box, and were returned to their cages after 5 min. On the third day, i.e., the training stage, 2 identical objects A were put into the test box, and the rats were given a 10-min period of unrestricted movement within the box. The total exploration time of the rats for the 2 objects A was recorded. On the fourth day, the rats were entered into the testing stage: a new object (object B) was used to randomly replace one of the objects A (old object), and rats were permitted entry into the test box to explore freely for 5 min. The time spent by the rats exploring old (A) and new objects (B) was recorded. The discrimination index (DI) of the rats was analyzed in the detection stage as follows: DI = (time spent exploring object B—time spent exploring object A)/(time spent exploring object B + time spent exploring object A) × 100%. To prevent the odor from the preceding rat from affecting the experimental results, the test box was cleaned with 75% alcohol prior to each rat being tested. The definition of object exploration is when the rats engage in active exploration of the objects by touching them with their nose at a distance of 2 cm or less.

### Statistical analysis

Statistical analysis was conducted using SPSS *v*26.0. One-way analysis of variance was utilized to compare several means, and the data were presented as mean ± standard deviation. The least significant difference test was used to assess homogeneous variance, and the Tamhane T2 test was used to evaluate uneven variance. A *P*-value < 0.05 was considered statistically significant.

## Results

### Pyroptosis and ferroptosis jointly participate in the neuronal-damage process after SE

**Fig. 1:**
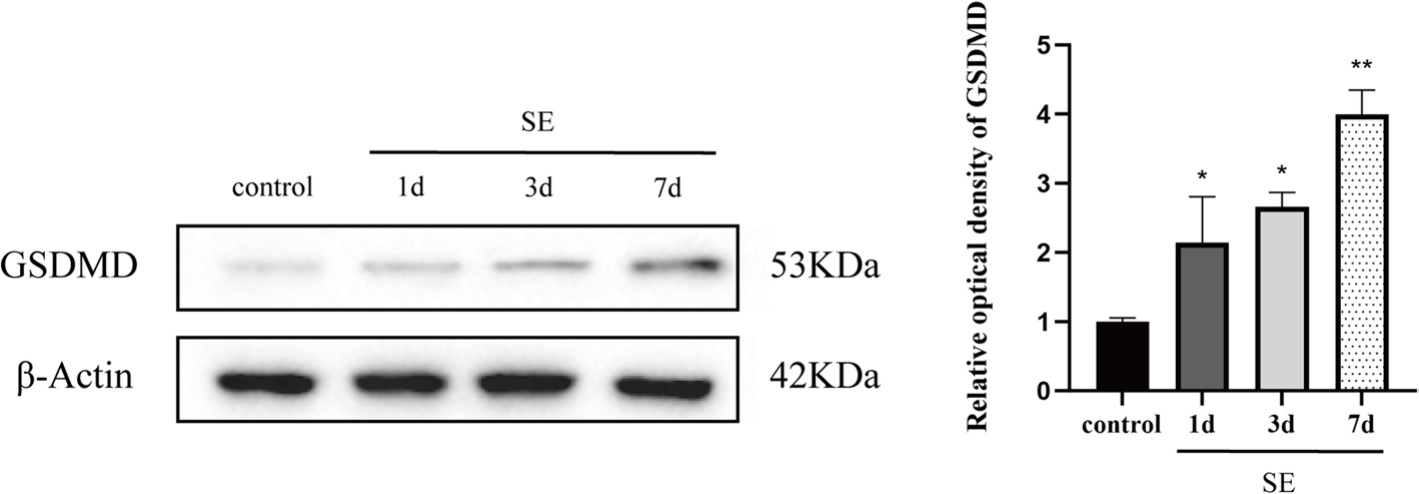
GSDMD expression in the rat hippocampus in the control and SE groups, as detected using western blotting. GSDMD expression increased on day 1 and further increased on days 3 and 7 after SE. *n* = 3 in each group. The data were obtained using densitometry and normalized using β-Actin. *P < 0.05, **P < 0.01, compared with the control group. GSDMD, Gasdermin D; SE, status epilepticus

**Fig. 2:**
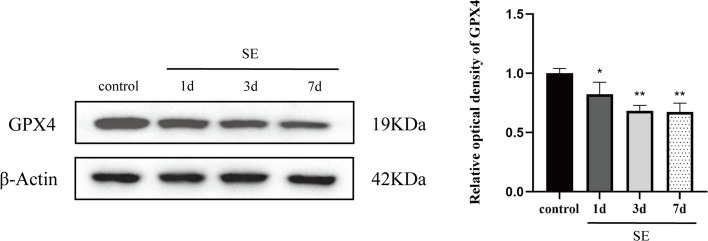
GPX4 expression in the rat hippocampus in the control and SE groups, as detected using western blotting. GPX4 expression progressively decreased on days 1, 3, and 7 after SE. The data were obtained using densitometry and normalized using β-Actin. *n* = 3 in each group. *P < 0.05, **P < 0.01, compared with the control group. GPX4, glutathione peroxidase; SE, status epilepticus

We performed Western blotting to assess the expression levels of GSDMD and GPX4 proteins in the hippocampus, aiming to explore the potential relationship between pyroptosis and ferroptosis in the context of epilepsy. Hippocampus from SE rats were collected 1, 3, and 7 days after Li-Pilo injection. The results indicated that the expression of GSDMD was upregulated after the induction of SE, while that of GPX4 was downregulated after SE. Compared with the control group, the SE group showed an increase in GSDMD expression on day 1, and further increases in GSDMD expression on days 3 and 7 (*P* = 0.030, 0.010, and 0.000, respectively; Fig. 1). In contrast, the expression of GPX4 progressively decreased on days 1, 3, and 7 in the SE group (*P* = 0.015, 0.001, and 0.000, respectively; Fig. 2). These findings suggest that pyroptosis and ferroptosis are jointly involved in the neuronal-damage process after SE.

### GSDMD knockdown inhibits neuronal damage after SE

**Fig. 3:**
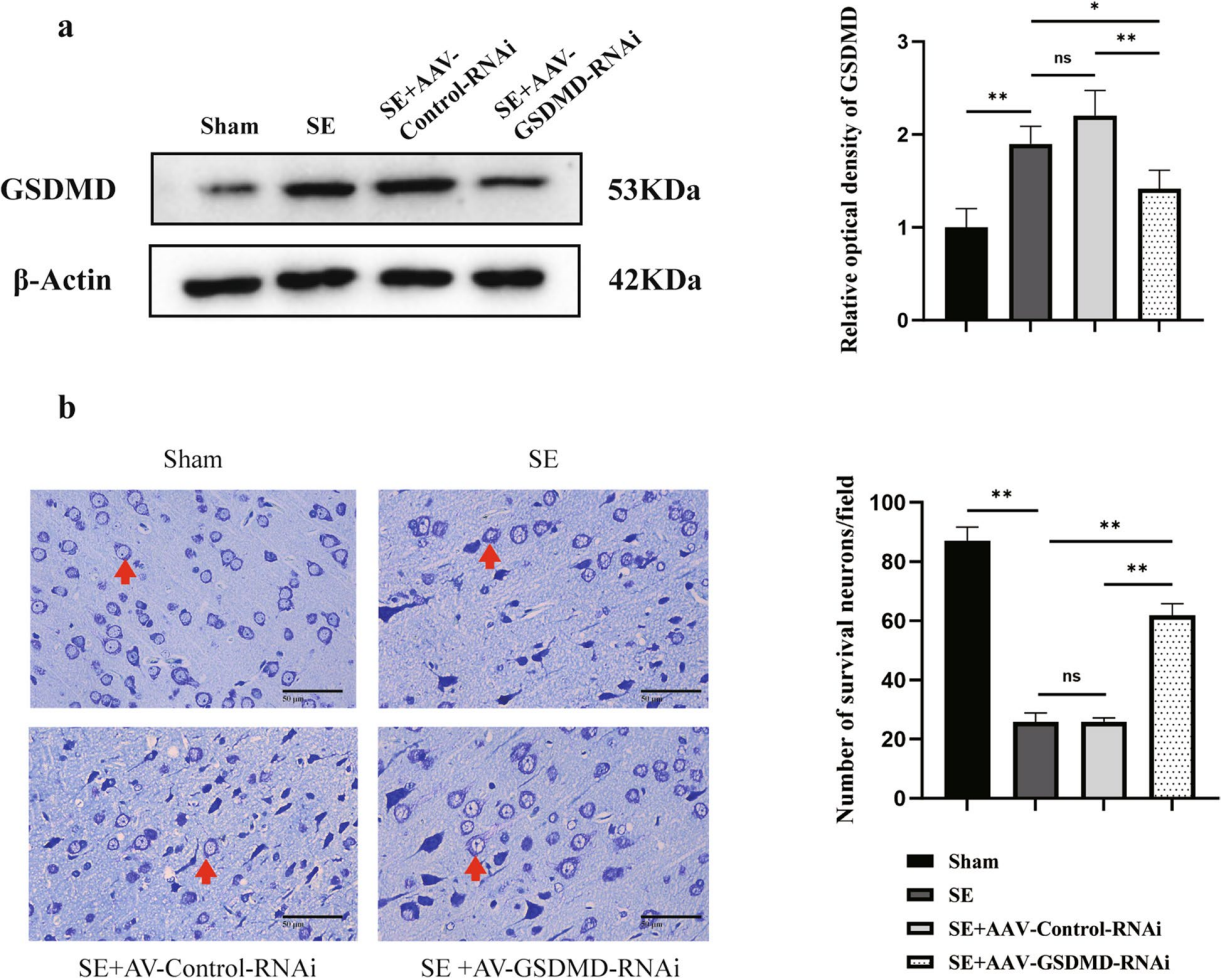
The knockdown of GSDMD protected hippocampal neurons in Li-Pilo-induced SE rats. **a** Validation of GSDMD Knockdown by western blotting. **b** Nissl staining in subregions of the hippocampus indicated an increased number of viable neurons in the SE + AAV-GSDMD-RNAi group (original magnification, × 400). The red arrowheads indicate Nissl bodies. Scale bar = 50 μm. Statistical results show that GSDMD knockdown increases the number of viable cells. *n* = 3 in each group. Data are presented as mean ± standard deviation. *P < 0.05, **P < 0.01, ns represents no significance. GSDMD, Gasdermin D; Li-Pilo, lithium-pilocarpine; SE, status epilepticus; AAV, adeno-associated virus; RNAi, RNA interference

Considering the notable increase in GSDMD expression in the hippocampus of the SE rats, we next knocked down GSDMD by injecting AAV-GSDMD-RNAi directly into the hippocampus. The knockdown efficiency of GSDMD was validated by western blotting. Representative blots in Fig. 3a demonstrate a substantial reduction of GSDMD protein in the SE + AAV-GSDMD-RNAi group relative to the SE + AAV-Control-RNAi group (*P* = 0.002). And then Nissl staining was used to determine whether the post-SE neuronal damage was reduced. We found that the SE group demonstrated a significantly higher incidence of neuronal death compared to the sham group (*P* = 0.000). Importantly, administration of AAV-GSDMD-RNAi markedly attenuated neuronal death in SE + AAV-GSDMD-RNAi rats as compared to the sham group (*P* = 0.000), while no such effect was observed in rats treated with AAV-Control-RNAi (*P* = 0.969). Thus, GSDMD knockdown attenuated SE-induced neuronal damage in the hippocampus (Fig. 3b).

### GSDMD knockdown inhibits pyroptosis

**Fig. 4:**
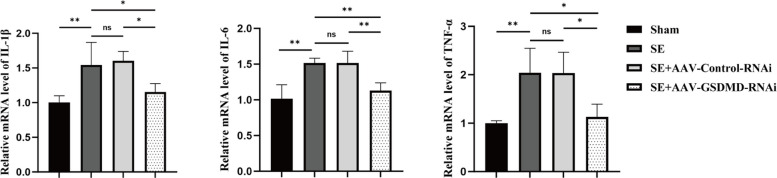
mRNA expression levels of IL-1β, IL-6, and TNF-α in different groups. *n* = 3 in each group. Data are presented as mean ± standard deviation. *P < 0.05, **P < 0.01, ns represents no significance. IL, interleukin; TNF-α, tumor necrosis factor-α; GSDMD, Gasdermin D; AAV, adeno-associated virus; RNAi, RNA interference

To investigate the impact of GSDMD knockdown on pyroptosis in SE, We measured the mRNA expression levels of the pyroptosis-related cytokines IL-1β, IL-6, and TNF-α using qRT-PCR (Fig. 4). The findings demonstrated a significant rise in the mRNA expression levels of IL-1β, IL-6, and TNF-α in the SE group as compared to the sham group (*P* = 0.009, 0.003, and 0.007, respectively). Compared with the SE group and SE + AAV-Control-RNAi group, the SE + AAV-GSDMD-RNAi group showed a significant decrease in the mRNA expression levels of IL-1β (*P* = 0.038 vs SE,* P* = 0.021 vs Control-RNAi), IL-6 (*P* = 0.010 vs SE,* P* = 0.010 vs Control-RNAi), and TNF-α (*P* = 0.014 vs SE,* P* = 0.015 vs Control-RNAi). The SE + AAV-Control-RNAi group showed no statistically significant changes when compared to the SE group (*P* = 0.713, 1.000, and 0.982, respectively). Overall, the results indicated that in SE rats, the reduction of GSDMD expression significantly decreased the expression of the pyroptosis-related cytokines IL-1β, IL-6, and TNF-α, suggesting that inhibiting GSDMD attenuated pyroptosis activation to some extent after SE.

### GSDMD knockdown inhibits ferroptosis

**Fig. 5:**
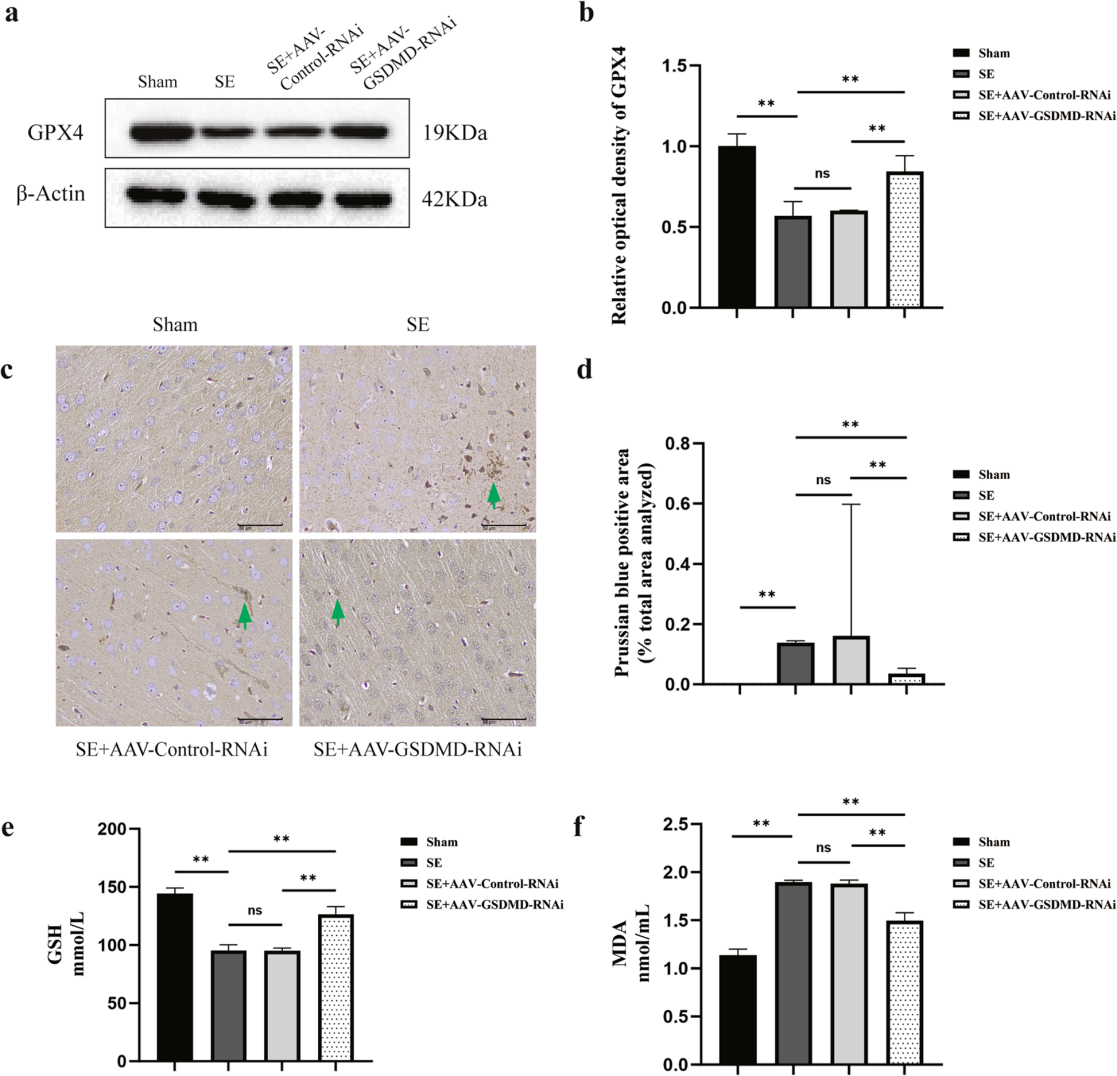
**a**, **b** Representative western blot samples and quantitative analysis of GPX4 in the hippocampus of the rats in each group. **c** Prussian blue staining of the hippocampus. The green arrowheads indicate positive expression. Scale bar = 50 μm. **d** Prussian blue-positive area as a percentage of the total area analyzed in each group. **e**, **f** ELISA to detect the expressions of GSH and MDA in the hippocampal tissue of rats. *n* = 3 in each group. Data are presented as mean ± standard deviation. *P < 0.05, **P < 0.01, ns represents no significance. GPX4, glutathione peroxidase 4; ELISA, enzyme-linked immunosorbent assay; GSH, reduced glutathione; MDA, malonaldehyde; GSDMD, Gasdermin D; SE, status epilepticus; AAV, adeno-associated virus; RNAi, RNA interference

Subsequently, we investigated whether the knockdown of GSDMD exerts any impact on ferroptosis following SE. We observed that GSDMD knockdown not only suppressed pyroptosis but also induced alterations in the ferroptosis pathway. We first measured the levels of the key ferroptosis enzyme GPX4. The findings demonstrated a considerable rise in the level of GPX4 in the SE + AAV-GSDMD-RNAi group, as compared to the SE group (*P* = 0.002) and SE + AAV-Control-RNAi group (*P* = 0.004); however, the level of GPX4 remained largely unchanged in the SE + AAV-Control-RNAi group as compared to the SE group (*P* = 0.623; Fig. 5a, b). Subsequently, Prussian blue staining revealed substantial increase in iron deposition in the hippocampus of the SE group compared to the sham group (*P* = 0.000). This suggests a significant alteration in iron homeostasis following SE. Moreover, GSDMD knockdown resulted in a clear decrease in iron deposition in the hippocampus, as compared to the SE group (*P* = 0.001) and SE + AAV-Control-RNAi group (*P* = 0.000). However, in the SE + AAV-Control-RNAi group, iron deposition showed no significant difference when compared to the SE group (*P* = 0.280; Fig. 5c, d). Ultimately, the levels of GSH and MDA expression were assessed through ELISA. GSH is a negative regulator of the ferroptosis pathway, while MDA is a metabolite of ferroptosis. The findings demonstrated a substantial rise in MDA (*P* = 0.000). Conversely, there was a pronounced decrease in GSH levels in the SE group, in contrast to the sham group (*P* = 0.000). After knocking down GSDMD, the levels of GSH were significantly upregulated as compared to the SE group (*P* = 0.000) and SE + AAV-Control-RNAi group (*P* = 0.000). Meanwhile, MDA levels showed a notable decrease relative to the SE group (*P* = 0.000) and SE + AAV-Control-RNAi group (*P* = 0.000). The levels of GSH and MDA showed no significant difference when comparing the SE + AAV-Control-RNAi group with the SE group (*P* = 0.932 and 0.696, respectively; Fig. 5e, f). Collectively, these findings suggest that the downregulation of GSDMD can upregulate GPX4 expression, attenuate iron deposition, enhance GSH levels, and reduce MDA levels, indicating the inhibition of the ferroptosis pathway.

### Cognitive and behavioral improvement in SE rats after GSDMD knockdown

**Fig. 6:**
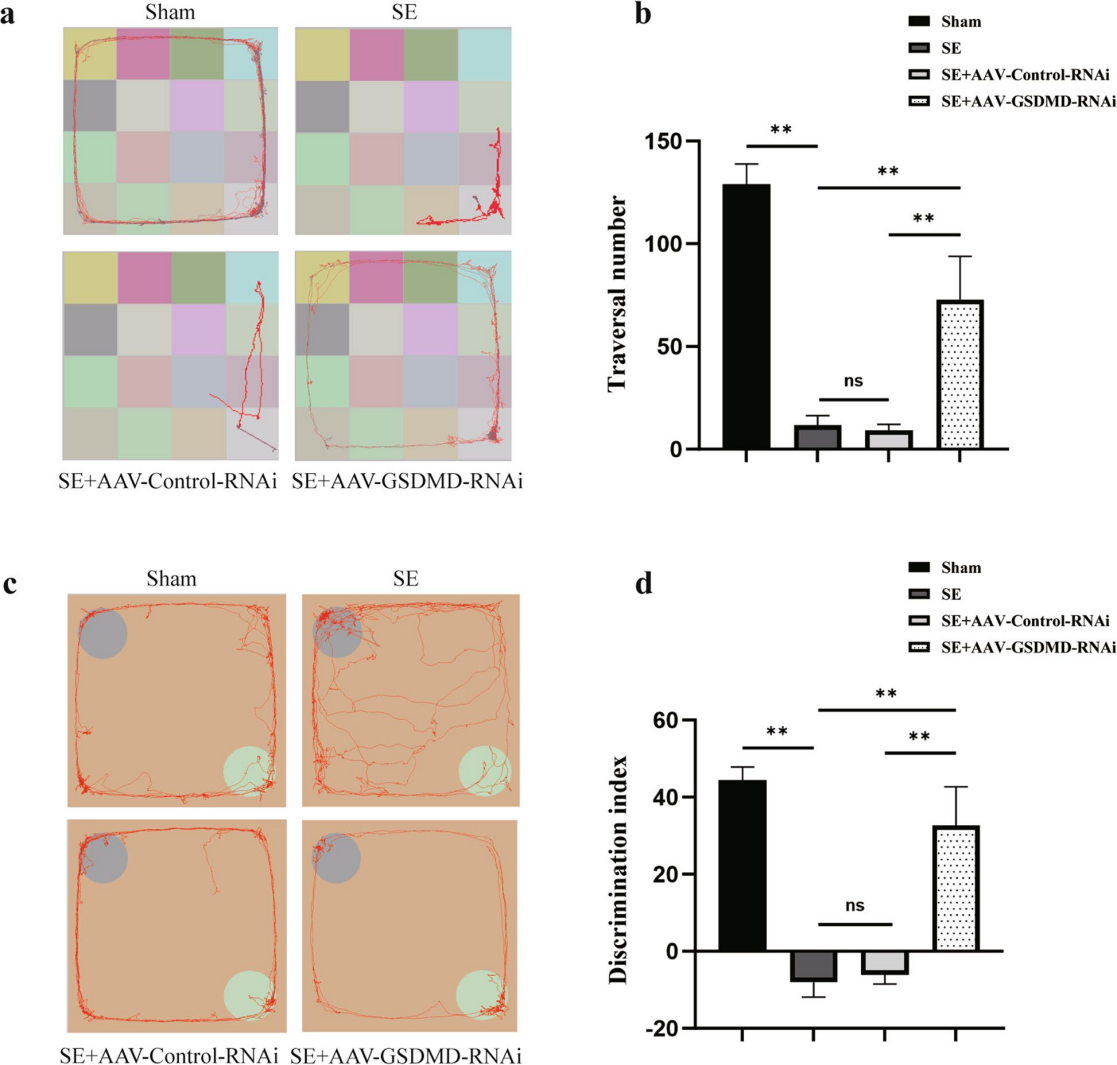
Inhibiting GSDMD alleviates the impairment of cognitive and behavioral functions in SE rats. **a** Representative route of rats during a spontaneous-activity test. The activity routes of the rats in the sham group and SE + AAV-GSDMD-RNAi group are distributed around the box. The activity routes of the rats in the SE group and SE + AAV-Control-RNAi group are limited to one side of the box. **b** Total number of grids crossed in the spontaneous-activity test. **c** Representative routes of rats in the novel object-recognition test. The blue circle in the northwest quadrant represents object A; the green circle in the southeast quadrant represents object B. The rats in the sham group and the SE + AAV-GSDMD-RNAi group spent more time being active around the new object B as compared to the old object A. The rats in the SE group and the SE + AAV-Control-RNAi group spent less time being active around the new object B as compared to the old object A. **d** Discrimination index in the novel object-recognition test. *n* = 4 in each group. Data are presented as mean ± standard deviation. *P < 0.05, **P < 0.01, ns represents no significance. GSDMD, Gasdermin D; SE, status epilepticus; AAV, adeno-associated virus; RNAi, RNA interference

Cognition and behavioral functions were assessed using two hippocampal-dependent learning and memory tasks, namely, the spontaneous-activity test and the novel object-recognition test. The results from the spontaneous-activity test revealed a significant reduction in grid crossings for the SE group when compared to the sham group (*P* = 0.000). However, these effects were partially reversed in the SE + AAV-GSDMD-RNAi group. After knocking down GSDMD, the number of crossed grids increased significantly as compared with the SE group (*P* = 0.000) and SE + AAV-Control-RNAi group (*P* = 0.000; Fig. 6a, b). The new object-recognition test revealed that the DI was significantly lower in the SE group than in the sham group (*P* = 0.000). Compared with the SE group and SE + AAV-Control-RNAi group, the SE + AAV-GSDMD-RNAi group showed a significantly increased DI (*P* = 0.000 and 0.000, respectively; Fig. 6c, d). In both experiments, no discernible disparity was observed between the rats in the SE + AAV-Control-RNAi group and the SE group (*P* = 0.798 and 0.682, respectively). The above results indicated severe impairment of cognitive and behavioral functions in SE rats, and that GSDMD knockdown improved both the cognitive and behavioral abilities of the rats.

## Discussion

The impact of short-term epileptic seizures on neurons is minimal, but SE can cause irreversible neuronal injury, especially in the hippocampus. The pathological characteristics of hippocampal injury include loss of neurons, gliosis, and remodeling of neuronal networks. This process has the potential to lead to the formation of new epileptic focus as well as impairment of cognitive and behavioral functions [18–20]. These changes also hinder the effect of anti-seizure medications and result in refractory epilepsy [21].

Our study confirmed that pyroptosis and ferroptosis participated in the process of neuronal loss concurrently in the hippocampus after SE. In classic pyroptosis pathway, GSDMD is cleaved by inflammasome-activated caspase-1, generating pore-forming activity that can directly perforate and rupture the cell membrane. Simultaneously, the precursors of IL-1β and IL-18 are cleaved into their mature forms by activated caspase-1, and then released into the extracellular environment through the pores created in the cell membrane to cause inflammatory reactions and lead to cell injury [22]. In addition, GPX4 serves as a crucial enzyme in the GSH-dependent pathway of ferroptosis, primarily involved in the reduction of cytotoxic lipid peroxides to their corresponding alcohols through the consumption of two molecules of GSH. It has a crucial function in decreasing the production of lipid ROS, and serves as a primary negative regulator of ferroptosis. Therefore, GSDMD and GPX4 are important markers of pyroptosis and ferroptosis, respectively. In this study, western blotting analysis revealed the significant upregulation of GSDMD expression and downregulation of GPX4 expression in the rat hippocampus following SE induced by Li-Pilo, suggesting the involvement of both pyroptosis and ferroptosis in neuronal loss after SE.

Nissl staining showed that after GSDMD knockdown, the quantity of neurons that survived following SE showed a significant increase, verifying that inhibiting pyroptosis can alleviate neuronal damage after SE. It has been suggested that pyroptosis promotes calcium influx during epileptic seizures by releasing inflammatory factors, rendering neurons highly sensitive to glutamic acid and N-methyl-d-aspartate-mediated excitotoxicity and oxidative stress and eventual neuronal death [23]. In our study, the levels of inflammatory factors following SE were measured using qRT-PCR. The results indicated a considerable rise in the mRNA expression levels of IL-1β, IL-6, and TNF-α in the hippocampus of rats following SE. By inhibiting GSDMD through AAV-GSDMD-RNAi, we found a notable decrease in the mRNA levels of IL-1β, IL-6, and TNF-α. Therefore, we speculate that inhibiting GSDMD can alleviate neuronal loss after SE by inhibiting pyroptosis and neuroinflammation.

Lipid peroxides (LOOH) are a source of the toxic aldehyde MDA, which is generated during lipid peroxidation in ferroptosis. GPX4 plays a crucial role in protecting cells from lipid peroxidation damage by utilizing two molecules of GSH to convert LOOH into less harmful alcohols (LOH). When GPX4 levels decrease, the conversion of LOOH to LOH is impaired, leading to an accumulation of MDA and increased oxidative stress. One of the hallmark features of ferroptosis is the excessive accumulation of iron. Ferrous ions can transfer electrons to hydrogen peroxide, generating hydroxyl radicals, which then undergo oxidative reactions with biological molecules such as lipids, leading to excessive production of ROS and ultimately destroying the integrity of the cell membrane. In this study, the findings demonstrated that knockdown of GSDMD also, to some extent, inhibited ferroptosis, as indicated by increased levels of GPX4 and GSH, as well as decreased iron deposition and MDA levels. Hence, our results suggested that the neuroprotective effect of GSDMD knockdown may be associated with the simultaneous downregulation of pyroptosis and ferroptosis in the hippocampus of rats following SE.

**Fig. 7:**
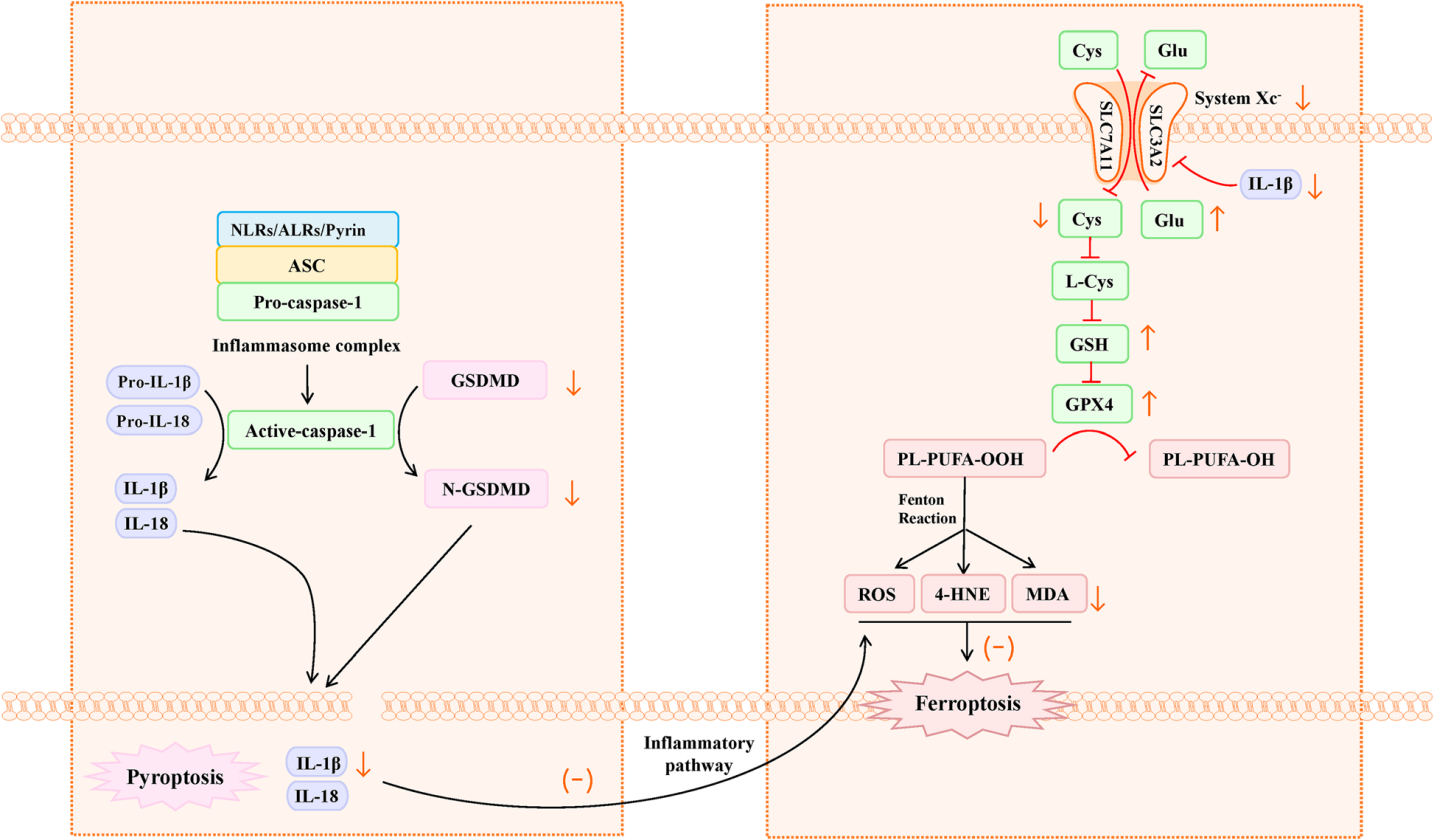
Inhibiting pyroptosis may suppress ferroptosis by inhibiting the inflammatory pathway. NLRs, nucleotide-binding and oligomerization domain-like receptors; ALRs, absent in melanoma 2 -like receptors; ASC, apoptosis-associated speck-like protein; Pro-IL-1β, the IL-1βprecursor; Pro-IL-18, the IL-18 precursor; IL-1β, interleukin-1β; IL-18, interleukin-18; GSDMD, Gasdermin D; PL-PUFA-OOH, lipid peroxides; PL-PUFA-OH, the corresponding alcohols of lipid peroxides; ROS, reactive oxygen species; 4-HNE, 4-hydroxy-2-nonenal; MDA, malondialdehyde; Cys, cystine; Glu, glutamate; GSH, glutathione; GPX4, Glutathione peroxidase 4

The interplay between pyroptosis and ferroptosis may be mediated by inflammatory responses. Accumulating evidence suggests that inflammatory factors are not only products of pyroptosis but also capable of inducing ferroptosis. Inflammasome activation and the subsequent release of inflammatory factors mediated by GSDMD constitute key steps in pyroptosis. Simultaneously, inflammasome activation and inflammatory factors can exacerbate intracellular oxidative stress and lipid peroxidation through diverse pathways, thereby inducing ferroptosis [24]. As demonstrated by earlier research, the activation of the NLRP3/JAK2/STAT3 pathway stimulates the production of downstream cytokines IL-1β and IL-18, which increases lipid peroxidation and imbalance of the antioxidant system, ultimately causing ferroptosis in asthma [25].Wu and colleagues also noted that the NLRP3-specific inhibitor MCC950 can effectively inhibit cerebral ischemia–reperfusion-mediated neuronal pyroptosis and reverse ferroptosis [26]. Luo et al. [27] observed that IL-1β can induce transcription of p53 in endothelial cells, downregulate the expression of xCT (the substrate-specific subunit of System Xc-), inhibit cystine uptake, and reduce GSH synthesis, ultimately enhancing ferroptosis in endothelial cells. Consequently, we speculate that GSDMD knockdown decreased inflammasome activation and release of inflammatory factors in the hippocampus, which subsequently downregulated ferroptosis (Fig. 7). This may represent an indirect regulatory mechanism.

It is reported that 30% to 40% of patients with epilepsy suffer from cognitive and behavioral dysfunction, which is characterized by memory and attention loss, decreased motor speed, language difficulties, and impaired executive function [28, 29]. Cognitive dysfunction significantly impacts the overall well-being and life quality of patients, and many patients perceive it to be more severe than epilepsy. Neuronal loss caused by SE in the hippocampus may interfere with the neural networks that underpin cognition, resulting in negative cognitive consequences [30]. In both Li-Pilo- and KA-induced SE animal models, a significant neurons loss in the hippocampus was observed, accompanied by cognitive impairment and chronic epileptic seizures [31–33]. This result was further corroborated by radiological and pathological studies of SE patients. In the subsequent monitoring of children who have experienced febrile SE, a trend toward delayed receptive language and motor development was found, which was related to acute hippocampal T2 hyperintensities on MRI [34]. In humans, following prolonged seizures, the hippocampus may first experience enlargement and hyperintense, eventually leading to atrophy. Neuropathological examination reveals substantial neuronal cell loss in the hippocampus [35, 36]. Hence, the rescue of hippocampal neurons is an important therapeutic approach to improve cognitive function after SE.

In recent years, many studies have inhibited pyroptosis by knocking down GSDMD, effectively improving the disease states. Xia et al. [37] first identified a gene set closely related to the pathogenesis of epilepsy through bioinformatics analysis. The results of correlation analysis showed that this gene set was highly correlated with the pyroptosis signaling pathway. Subsequently, a KA-induced SE mouse model was established, confirming that GSDMD-mediated pyroptosis was involved in KA-induced SE. Further experiments indicated that after using the GSDMD inhibitor dimethyl fumarate, astrocyte damage in the hippocampus was alleviated, the expression of inflammatory cytokines IL-1β and IL-18 was decreased, and recurrent spontaneous seizures at 7–21 days after SE were significantly decreased [38]. This finding supported the conclusion of our study. In psoriasis, knockdown of GSDMD has been confirmed to inhibit pyroptosis and alleviate psoriatic symptoms. Knockdown of GSDMD and treatment with disulfiram (a GSDMD inhibitor) can significantly inhibit pyroptosis in M5-induced HaCaT cells (an in vitro model of psoriasis), and simultaneously promote the apoptotic process. In the imiquimod-induced psoriasis-like mouse model, GSDMD knockdown suppressed pyroptosis and improved skin lesion severity, alleviating erythema, epidermal thickness, and inflammatory cell infiltration. Mechanistically, GSDMD knockdown inhibited the NLR pathway, accompanied by reduced protein levels of NLRP3, NOD1, NOD2, and PYCARD [39]. In addition, in diabetes-induced myocardial injury, it has been confirmed that NLRP3 gene knockdown can inhibit pyroptosis and ferroptosis. In the diabetic cardiomyopathy model, the myocardial fibers are loosely arranged, inflammatory cell infiltration is observed, and mitochondrial cristae rupture occurs. The expression of GSDMD-NT significantly increased in the diabetes group, while the protein expressions of xCT and GPX4 significantly decreased, suggesting that both pyroptosis and ferroptosis were involved in the occurrence of the disease. Knockdown of the NLRP3 gene can reverse the above changes, alleviate myocardial injury, and inhibit pyroptosis and ferroptosis. This indicates that regulating the inflammatory pathway can simultaneously modulate pyroptosis and ferroptosis, further supporting the results of our study [40].

In our study, pyroptosis and ferroptosis after SE caused neuronal loss in the hippocampus and resulted in compromised spontaneous activity, exploratory behavior and learning abilities of rats in the spontaneous-activity test and novel object-recognition test. However, GSDMD knockdown significantly improved cognitive and behavioral functions in SE rats by concurrently downregulating pyroptosis and ferroptosis in the hippocampus.

Recently, an increasing number of research findings indicate a reciprocal link between pyroptosis and ferroptosis. Zhu et al. [41] developed UCNP-Cro/FA nanoparticles that reduce Fe^3^⁺ to Fe^2^⁺, thereby inducing ferroptosis. Meanwhile, hydroxyl radicals generated during ferroptosis can damage the lysosomal membrane, increasing its permeability and releasing Fe^2^⁺ and cathepsin B, which simultaneously trigger Caspase-1/GSDMD-dependent pyroptosis. These results provide compelling evidence for a collaborative effect between pyroptosis and ferroptosis in the process of cell damage. Similarly, Tong et al. [42] demonstrated that the ferroptosis inhibitor LPT1 not only reduces ferroptosis markers (ACSL4, ALOX15) and lipid peroxidation products (4-HNE, MDA) in metabolic dysfunction-associated fatty liver disease, but also decreases pyroptosis indicators. However, the exact mechanism remains unclear. Recent studies support that there exists a regulatory network between pyroptosis and ferroptosis. This perspective aligns with our findings.

This study has several limitations. First, the precise interaction between pyroptosis and ferroptosis following SE remains unclear. Our research suggests that pyroptosis could play a role in regulating ferroptosis, but we are still unable to explain this finding with a direct regulatory mechanism. It is possible that the inflammatory and mTOR pathways indirectly contribute to this process, but this remains unconfirmed. Future research could investigate whether exogenous inflammatory cytokines reverse the protective effect of GSDMD knockdown on ferroptosis and assess the impact of GSDMD knockdown on the mTOR pathway and ferritinophagy. Further clarification of the relationship between pyroptosis and ferroptosis after SE may help identify new therapeutic targets for epilepsy and SE. Second, the sample size in this study is relatively small. Although the existing sample size is sufficient to demonstrate statistically significant effects and consistent results, larger studies are needed to enhance the reliability and generalizability of our findings. Finally, the translational applicability of our results should be considered cautiously. Although the Li-Pilo-induced SE model has been extensively validated, it does not fully replicate the complex pathological process of human SE. Therefore, the neuroprotective strategy targeting GSDMD needs validation in more clinically relevant models.

## Conclusions

In summary, this study found that both pyroptosis and ferroptosis may participate in the process of neuronal damage in Li-Pilo-induced SE rat model. In addition, knockdown of GSDMD was found to potentially modulate pyroptosis and ferroptosis, protect hippocampal neurons, and enhance cognitive function in rats subjected to SE. This finding offers a promising new therapeutic target for SE treatment.

## Supplementary Information

Supplementary Material 1.

## Data Availability

The datasets used and/or analysed during the current study are available from the corresponding author on reasonable request.

## Abbreviations

4-HNE: 4-Hydroxy-2-nonenal
AAV: Adeno-associated virus
DI: Discrimination index
ELISA: Enzyme-linked immunosorbent assay
GPX4: Glutathione peroxidase 4
GSH: Glutathione
GSDMD: Gasdermin D
IL: Interleukin
KA: Kainic acid
Li-Pilo: Lithium-pilocarpine
LOH: The corresponding alcohols of lipid peroxides
LOOH: Lipid peroxides
MDA: Malonaldehyde
mTOR: Mechanistic target of rapamycin
NLRP1: NLR family pyrin domain containing 1
NLRP3: NLR family pyrin domain containing 3
OD: Optical density
PVDF: Polyvinylidene fluoride
qRT-PCR: Quantitative reverse transcription polymerase chain reaction
RCD: Regulated cell death
ROS: Reactive oxygen species
SE: Status epilepticus
System Xc-: Cystine/glutamate reverse transporter
TLE: Temporal lobe epilepsy
TNF-α: Tumor necrosis factor-α
