# Supplementary material for: Knockdown of Gasdermin D protects hippocampal neurons by regulating both pyroptosis and ferroptosis in a rat model of status epilepticus
Source: Acta Epileptol. 2026 May 4;8:20. doi: 10.1186/s42494-026-00255-5 (PMC13137726; doi:10.1186/s42494-026-00255-5)
Supplement: Supplementary file 1 — Supplementary Material 1. [file 42494_2026_255_MOESM1_ESM.pdf]

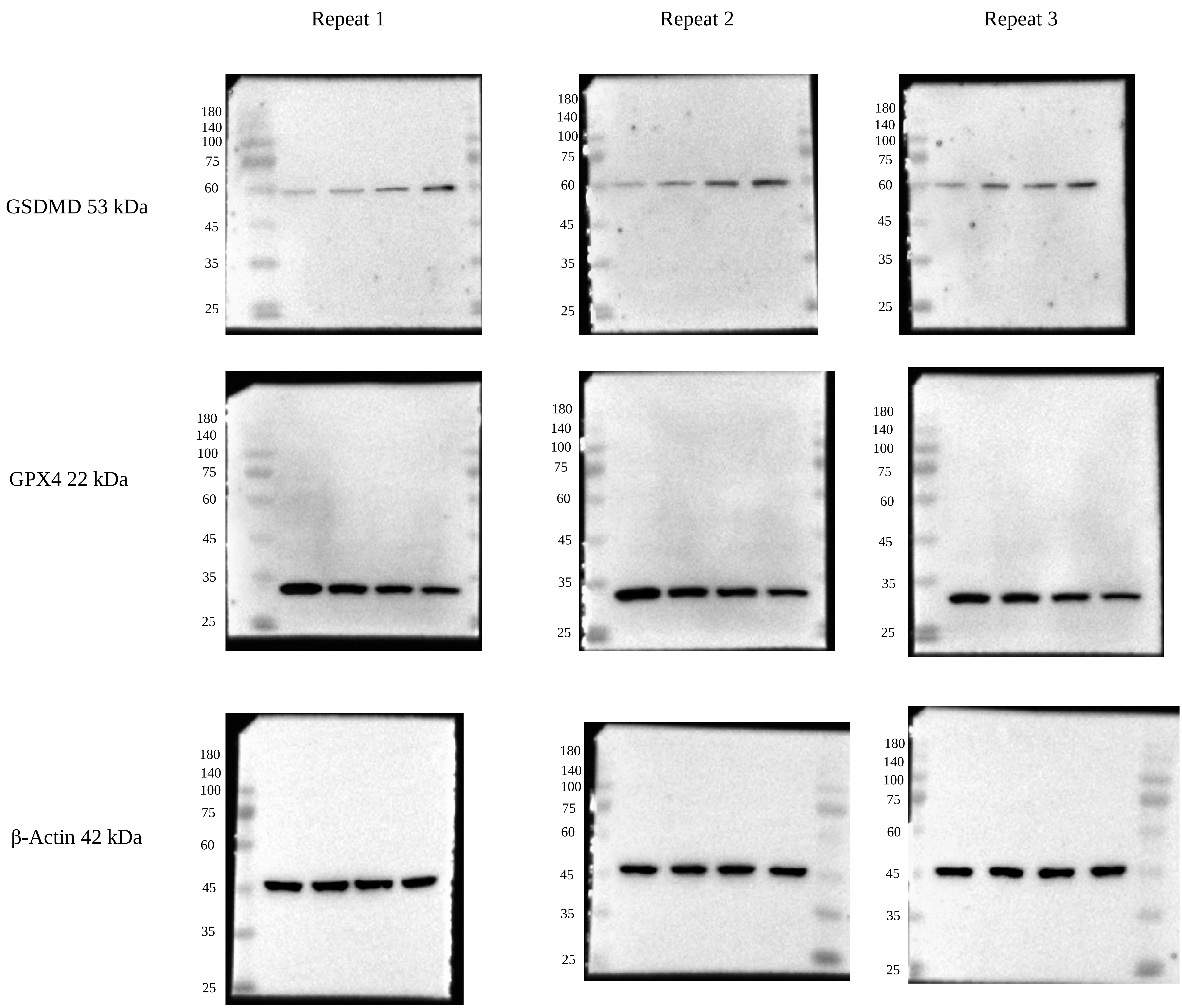

Repeat 1
Repeat 2
Repeat 3
GSDMD 53 kDa
GPX4 22 kDa
β-Actin 42 kDa
180
140
100
75
60
45
35
25

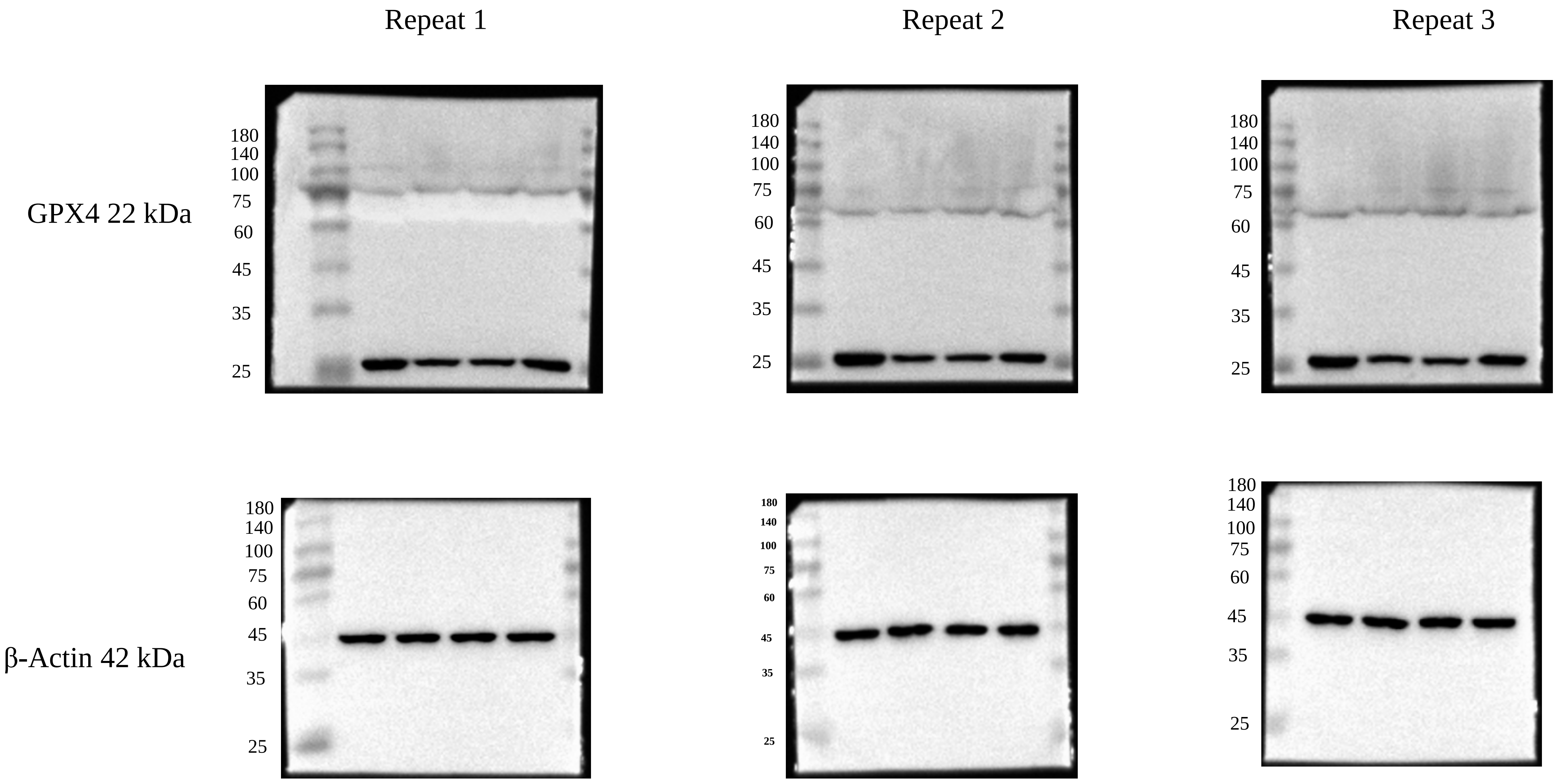
Repeat 1
Repeat 2
Repeat 3
GPX4 22 kDa
β-Actin 42 kDa
180
140
100
75
60
45
35
25
